# Paeoniflorin prevents postoperative peritoneal adhesion formation in an experimental rat model

**DOI:** 10.18632/oncotarget.21333

**Published:** 2017-09-28

**Authors:** Qi Gao, Guangbing Wei, Yunhua Wu, Na Yao, Cancan Zhou, Kai Wang, Kang Wang, Xuejun Sun, Xuqi Li

**Affiliations:** ^1^ Department of General Surgery, The First Affiliated Hospital of Xi’an Jiaotong University, Xi’an 710061, Shaanxi, China; ^2^ College of Nursing, Shaanxi University of Chinese Medicine, Xianyang 712046, Shaanxi, China; ^3^ Department of Hepatobiliary Surgery, The First Affiliated Hospital of Xi’an Jiaotong University, Xi’an 710061, Shaanxi, China

**Keywords:** peritoneal adhesion, oxidative stress, inflammation, paeoniflorin, postoperation

## Abstract

Although materials and modern surgical techniques have been developed to suppress postoperative adhesions, adhesion formation can still occur, and thus, a novel effective anti-adhesion drug is greatly needed. In the present study, we explored the efficacy of paeoniflorin treatment against postoperative peritoneal adhesions and examined the anti-oxidative stress and anti-inflammatory properties of PE. Forty-eight male Sprague-Dawley rats were randomly divided into 6 groups for the study: the sham, control, hyaluronan and three concentrations (10, 20 and 40 mg/kg/d) paeoniflorin groups. Abdominal adhesions were created by abrasion of the caecum and its opposite abdominal wall. In the paeoniflorin groups, the rats were administered daily oral doses of paeoniflorin for 7 days. The abdominal cavities of the rats were reopened with a U-shaped incision to macroscopically grade the adhesions. Histologic analysis was performed, and oxidative stress, inflammatory cytokine, collagen fiber degradation and cytokeratin levels were measured. Macroscopic and histopathological measurements revealed that paeoniflorin reduced peritoneal adhesion and inflammation. Notably, treatment with paeoniflorin reduced the protein levels of TGF-β1, IL-6 and COX-2. The collagen fiber fractions were distinctly lower in the PE groups than in the control group. Western blotting analyses showed that paeoniflorin increased MMP-9 and superoxide dismutase-2 protein expression and sharply reduced α-SMA and COX-2 protein expression. Peritoneal mesothelium cells were more continuous and complete in animals treated with paeoniflorin. Our study suggests that paeoniflorin can be used to ameliorate peritoneal adhesions via anti-oxidative stress and anti-inflammatory actions during the postoperative period.

## INTRODUCTION

The incidence of adhesion formation is greater than 93% after an abdominal surgical procedure [1, 2]. However, few clinical doctors focus on the high incidence of peritoneal adhesion formation [3, 4] or complications, including bowel obstruction, infertility, pelvic pain and other serious conditions [5, 6]. Multiple products for the prevention of peritoneal adhesions have been marketed [7–10], including barrier materials. The prophylactic effects of these products are limited, and no intervention has been accepted as a criterion in the clinic [11–13].

Peritoneal adhesion is triggered immediately after the peritoneum undergoes surgery, trauma, infection or oxidative stress [14]. Trauma, surgery and injury induce peritoneal mesothelial cell dysfunction and result in fibrinogen-rich severe extravasation, which is then degraded via the process of fibrinolysis. When fibrinolysis is insufficient in the damaged peritoneum, adhesion formation develops due to the incomplete resorption of fibrinous deposits. Furthermore, an inflammatory response is also the result of peritoneal adhesion, in addition to dysregulated fibrinolysis and collagen production [15, 16]. Studies [17, 18] in rat models have shown that the infiltration of macrophages aggravates adhesion formation by the systematic depletion of macrophages themselves.

Paeoniflorin (PE) is the main active compound in peonies, which are used in traditional Chinese medicine [19]. The pharmacological effects of PE have been demonstrated in previous investigations. In rat models of rheumatoid arthritis [20], PE has been shown to play numerous roles, including the mitigation of oxygen-radical damage, anti-inflammatory actions and the elimination of neurotoxicity. Experimental rat models [20] of periodontitis have indicated that PE exerts its effects by decreasing the levels of inflammatory cytokines, including tumor necrosis factor-α (TNF-α), interleukin-β1 (IL-β1) and interleukin-6 (IL-6), by down-regulating the expression of cyclooxygenase-2 (COX-2), metal matrix proteinase-9 (MMP-9) and inducible nitric oxide synthase (iNOS) proteins [21]. In addition, PE significantly decreases the formation of intracellular reactive oxygen species (ROS) and increases the levels of superoxide dismutase (SOD), glutathione (GSH) and endogenous anti-oxidants at a cellular level [22].

Nonetheless, research on role of PE in the prevention of peritoneum adhesion formation has not yet been reported. The objective of the present research was to study the effectiveness of PE, a compound that has been shown to have anti-inflammatory and anti-oxidative effects, in the prevention of peritoneum adhesion formation in a rat model.

## RESULTS

### PE significantly reduces macroscopic peritoneal adhesion scores in a rat model

**Figure 1:**
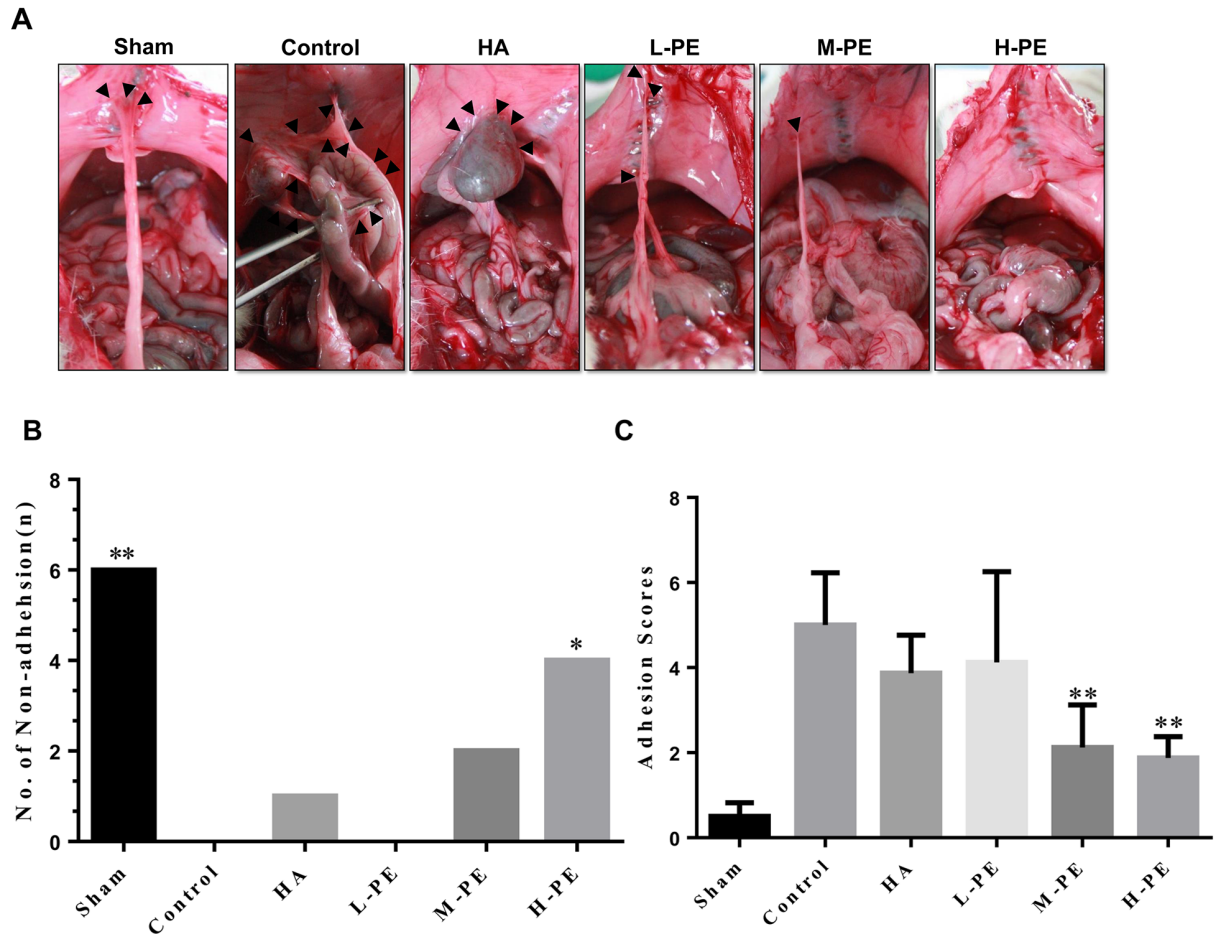
The administration of different concentrations of PE and sodium HA prevented postoperative abdominal adhesion formation in this rat model **(A)** The most representative images that showed the postoperative peritoneal adhesions (black triangle) in each groups of rats were chosen. In the sham operation group, almost no adhesions or only a small amount of peritoneal adhesions was present in response to the abdominal incision. In the control group, large adhesions formed in the abdominal cavity, and the adhesion tissue was strong and difficult to separate. In the HA group, moderate abdominal adhesion severity was observed, and the adhesion area was larger than that of the control group. The less significant adhesions in the L-PE group of rats were similar to those in the HA group in magnitude. There were only relatively soft adhesions in some rats in the M-PE and H-PE groups. The number of non-adhesions in each group **(B)**. The adhesion score of the groups **(C)**. ^*^Relative to the control group, *P*<0.05 and ^**^P<0.001.

All the rats survived to 7 days after the operation, and no significant differences were observed in the body weights of the rats among the six groups (Supplementary Figure 1). The adhesion results are shown in Figure 1A, 1B. The extent and severity of adhesion in the PE groups were less than those in the control group (*P*<0.05) (Supplementary Tables 1 and 2). The adhesion scores of the M-PE and H-PE groups were significantly lower than those of the control group (*P*<0.05) (Figure 1C).

The degree of peritoneal adhesion in each group was lower than that in the control group (Figure 1A). In the sham group, there was only a small amount of adhesion near the peritoneal incision, and intestine-to-abdominal-wall adhesions were relatively uncommon. In contrast, the control group exhibited numerous omentum-to-bowel, bowel-to-parietal peritoneum, parietal peritoneum-to-omentum, and bowel-to-bowel adhesions. The three groups of rats that had been treated with PE had fewer peritoneal adhesions than the control group. In the animals of the M-PE and H-PE groups, there was a low degree of adhesion formation, which appeared loose, and there was thin adhesion around the incision. The degree of adhesion in the HA group was better than that in the control group.

### PE reduces inflammatory cell infiltration, per histopathological examinations

**Figure 2:**
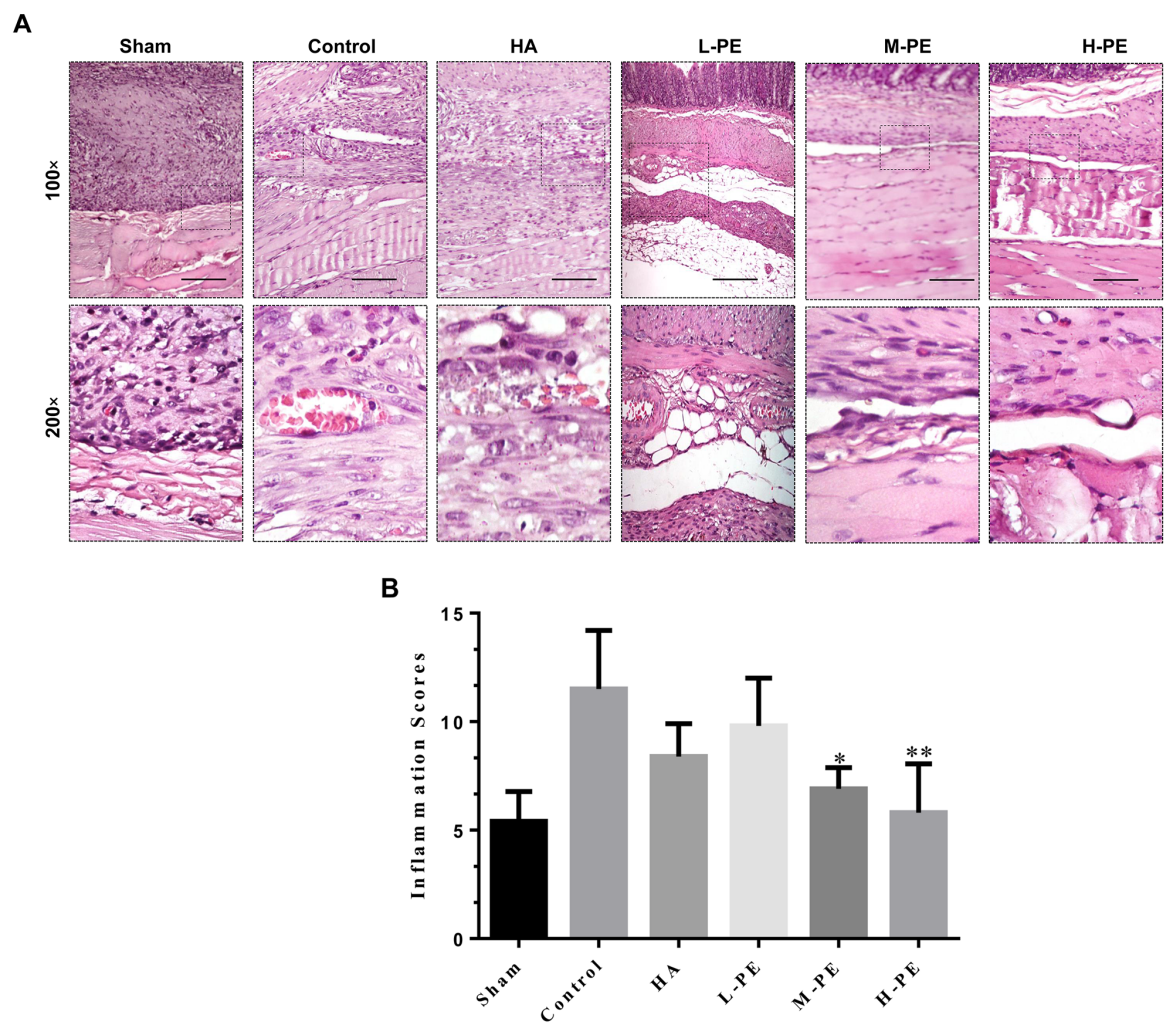
Inflammatory cell infiltration in postoperative peritoneal adhesions or the damaged areas on the opposing parietal peritoneum in each group of rats (n=8) **(A)** Representative images of HE staining in the postoperative peritoneal adhesion tissue or the damaged areas on the opposing parietal peritoneum in each group of rats (100× magnification in the upper row, 200× magnification in the lower row; scale bar represents 100 μm). **(B)** Inflammatory cell infiltration scores for the intra-abdominal adhesive tissue in each group of rats. ^*^ Relative to the control group, *P*<0.05 and ^**^P<0.001.

The histopathological examinations revealed that PE treatment reduced microscopic changes (Figure 2A), such as the increase in inflammatory cells as a result of postoperative peritoneal adhesion, and caused a notable decrease in the histopathological scores relative to the control groups (*P*<0.05) (Figure 2B). The histopathological scores of the three PE groups were different. The results revealed that the degree of inflammatory cell infiltration and the scores was associated with drug dose. Relative to the PE group, more inflammatory cell infiltration, granulation formation, and increased collagen deposition on the damaged parietal peritoneum and cecal specimens were observed in the control group. The H-PE group contained the fewest number of adhesions and had an optimal score (*P*<0.001).

### PE decreases inflammatory reaction and oxidative stress reaction

**Figure 3:**
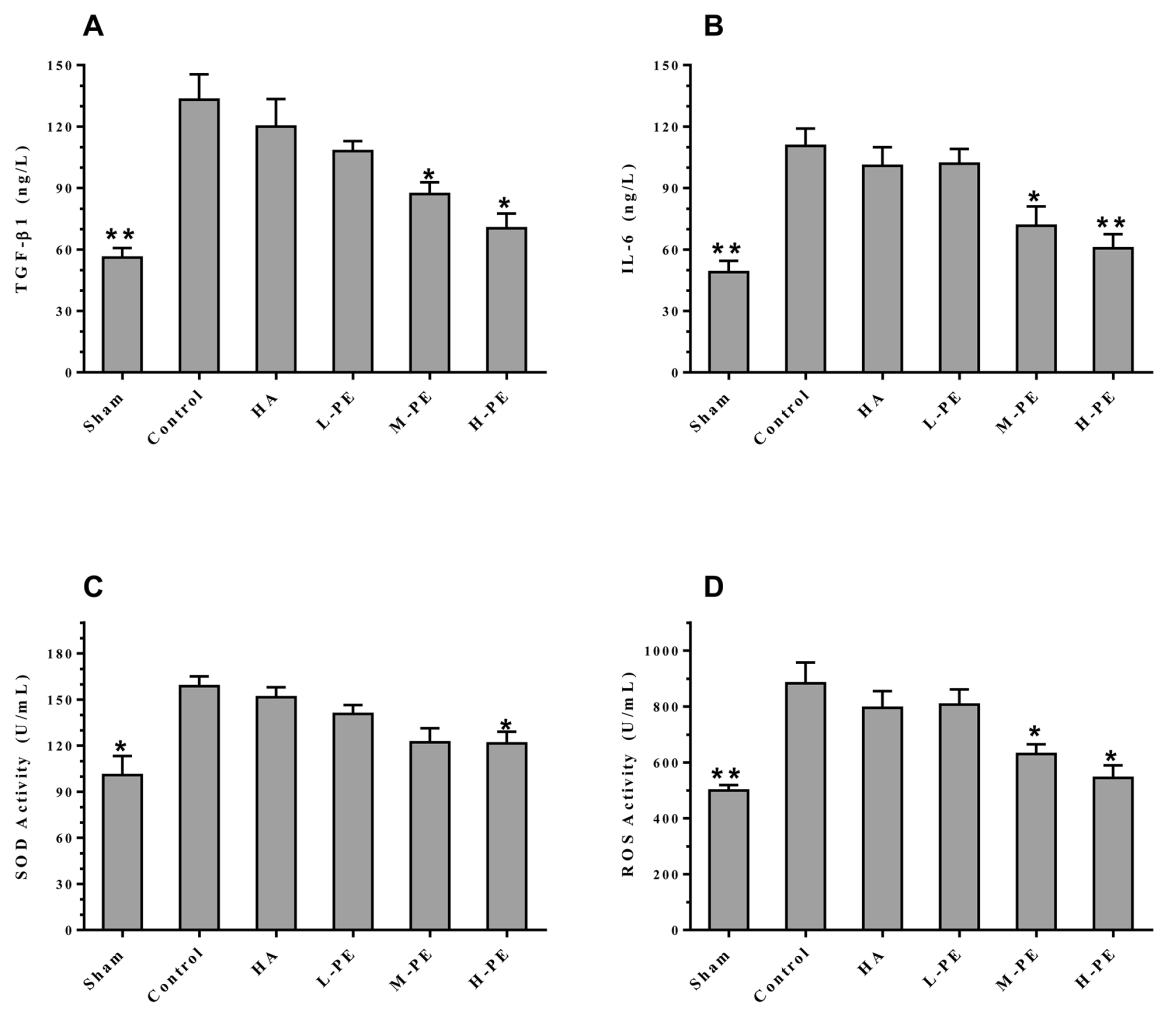
Inflammatory factors from arterial blood were quantified by ELISA The values (ANOVA) in the L-PE and M-PE groups were compared with the values in the control group **(A and B)**. The abdominal exudatives of SOD in the H-PE group **(C)** and the ROS in the M-PE and H-PE groups **(D)** were significantly lower than in the control group. ^*^ Relative to the control group, *P*<0.05, ^**^P<0.001.

**Figure 4:**
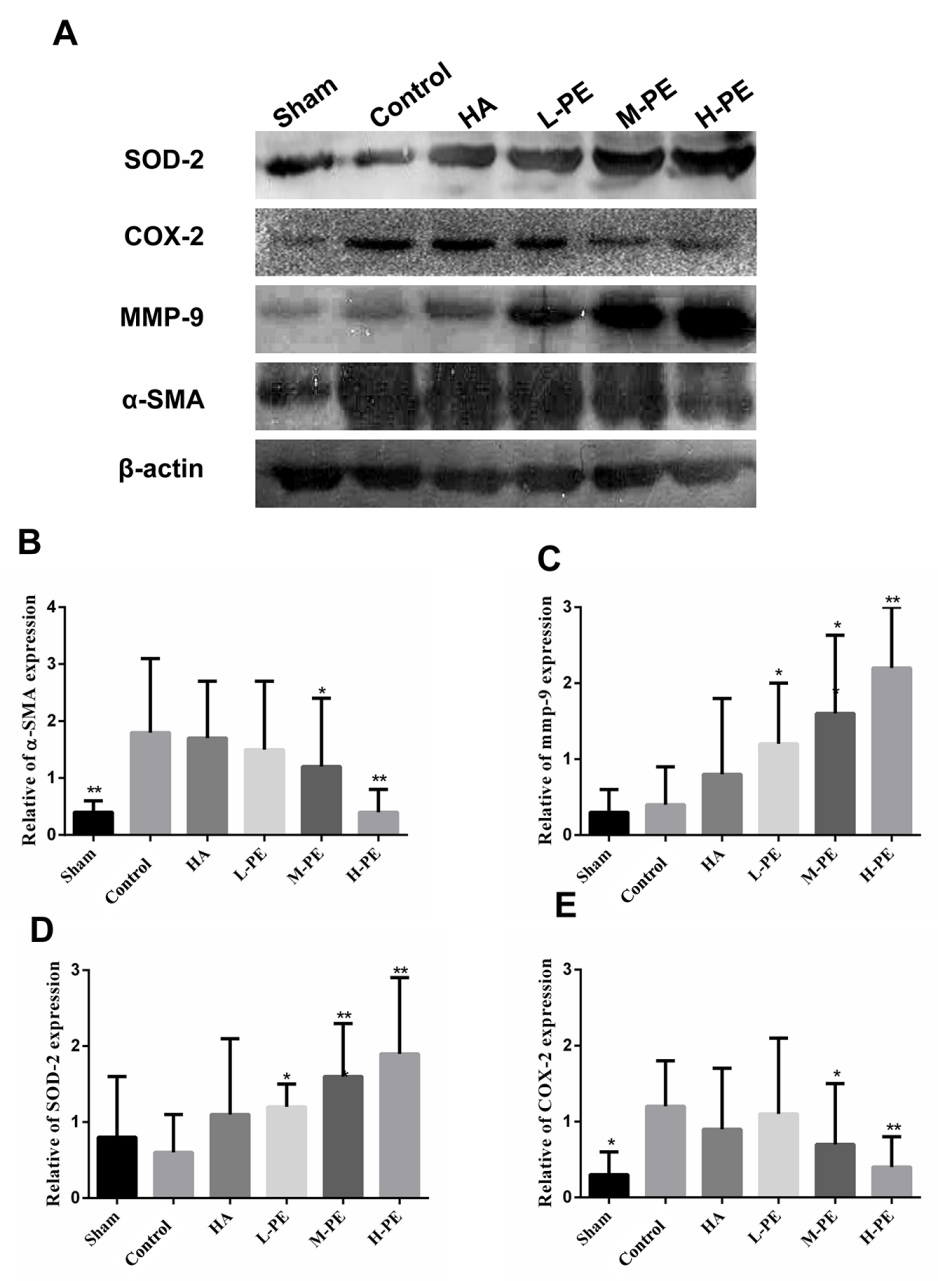
Expression of phosphorylated SOD-2, COX-2, MMP-9 and α-SMA proteins in the peritoneal adhesions or the damaged areas on the opposing parietal peritoneum was detected by western blotting **(A).** The relative expression of α-SMA, MMP-9, COX-2 and SOD-2 in different groups **(B, C, D and E)**. ^*^ Relative to the control group, *P*<0.05 and ^**^P<0.001.

**Figure 5:**
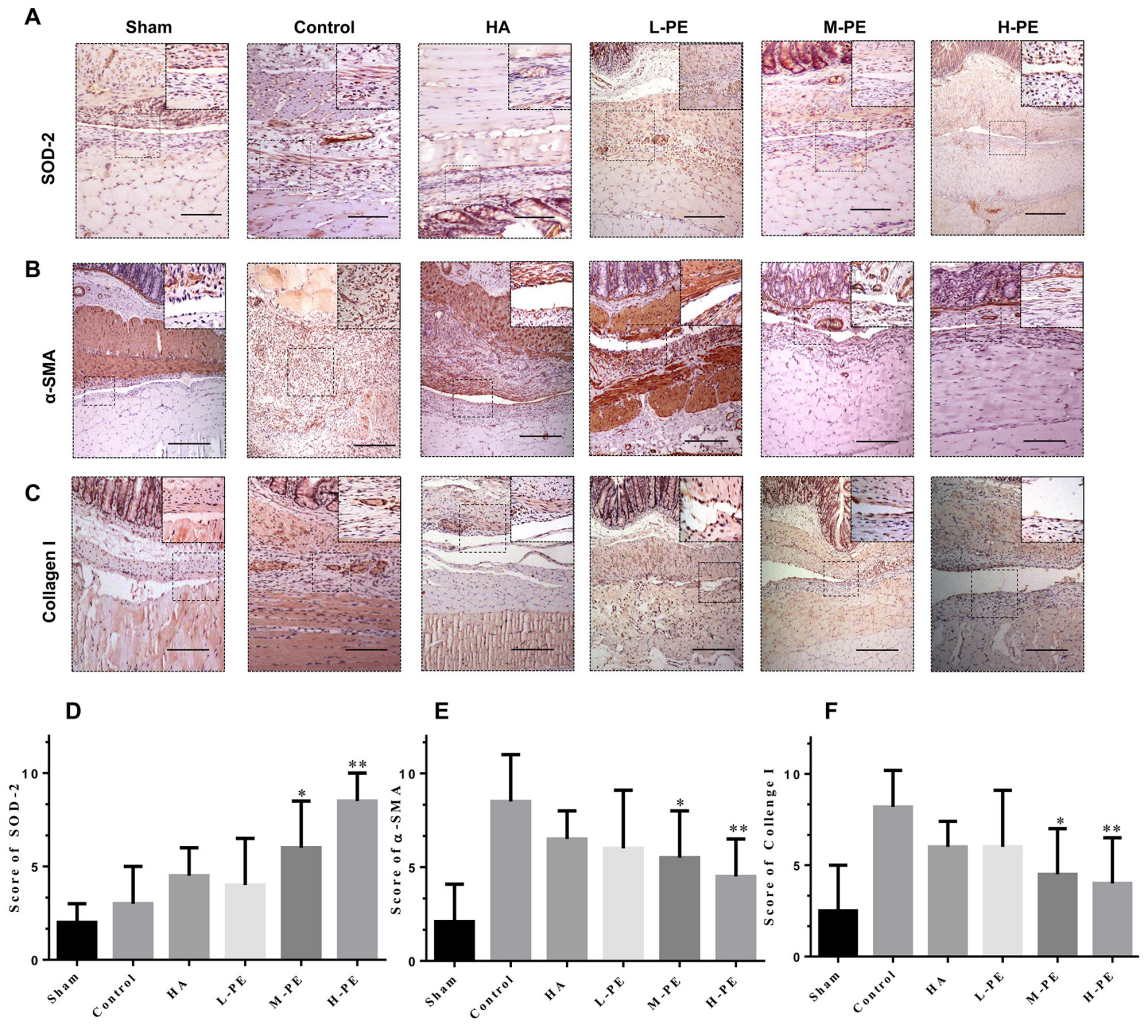
Immunohistochemical staining of SOD-2, α-SMA and collagen I in the postoperative peritoneal adhesions or the damaged areas on the opposing parietal peritoneum in each group of rats (100× magnification in the upper row, 200× magnification in the upper left corner) Relative to the sham group, the severity of adhesions was lower, and the higher the SOD-2 expression, the stronger the anti-oxidative stress activity **(A)**. With the increase in drug concentration, α-SMA **(B)** and collagen I **(C)** staining was gradually reduced, and the formation of fibers was gradually decreased. The black scale bar represents 100 μm. **(D, E and F**) show the SOD-2, α-SMA and collagen I staining scores in the different groups. ^*^ Relative to the control group, *P*<0.05 and ^**^P<0.001.

Relative to the control, PE reduced serum inflammatory cytokine levels. TGF-β1 and IL-6 were decreased in the M-PE and H-PE groups (*P*<0.05) (Figure 3A, 3B). In the enterocoelic fluid samples, the SOD in the H-PE group and ROS in both the M-PE and H-PE groups were reduced (*P*<0.05) (Figure 3C, 3D). Western blotting analysis revealed that the expression of COX-2 in rat peritoneal adhesion tissue was decreased after treatment with the PE. The M-PE and H-PE groups exhibited large decreases relative to the control and HA groups (Figure 4). The oxidative stress marker SOD-2 was increased in tissue specimens from the PE treatment groups. The results from the parallel testing of SOD-2 content in our specimens were consistent with those from immunohistochemical staining (Figure 5A, 5D) and western blotting (Figure 4) analyses in the PE groups.

### PE inhibits fiber development in the damaged peritoneum and/or adhesion tissues

When the α-SMA concentration was detected via immunohistochemical staining, a significant difference was observed between the assay groups and the control group (Figure 5B, 5E). In the control group, an abundance of spindle fibroblasts with positive brown staining was easily observed in the thick adhesive tissue. However, the sham group exhibited little or no positive staining. In the HA and L-PE groups, the number of fibroblasts with positive α-SMA expression was slightly lower than the number in the control group. Nevertheless, in the M-PE and H-PE groups, the prevalence of fibers was significantly decreased in the adhesion tissues and/or damaged peritoneum. The expression of α-SMA as detected by western blotting was also significantly different (Figure 4). Furthermore, we evaluated collagen I expression by immunohistochemical staining, which revealed that the deposition transitioned from high to low with the increase in PE doses (Figure 5C, 5F). Collagen I deposition in the HA group was the same as in the L-PE group. Therefore, our research shows that PE can inhibit the fibrosis of the damaged or adhesive peritoneum with increasing drug concentration in a rat model.

### Administration of PE decreases collagen exposition in the impaired peritoneum

**Figure 6:**
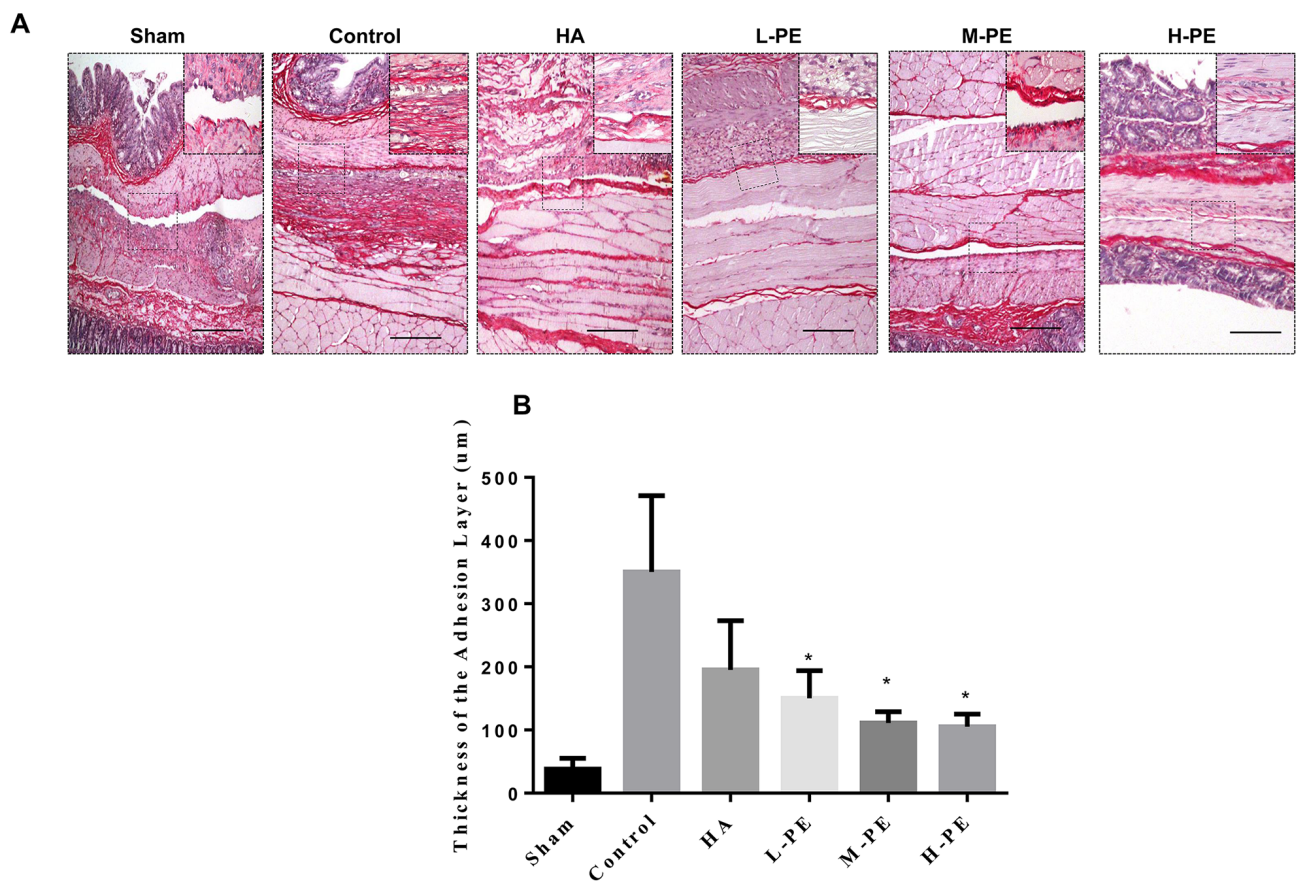
Picrosirius red staining revealed the presence of fibrosis in the postoperative peritoneal adhesions or in the damaged areas on the opposing parietal peritoneum in each group of rats (n=8) **(A)** Representative images of picrosirius red staining (100×; insets, 200×) in each group. **(B)** The collagen deposit thickness in the adhesive tissue of each group of rats was reduced relative to the control group. ^*^Relative to the control, *P*<0.05.

Collagen fibers were stained red via picrosirius red staining (Figure 6A). There was no collagen fiber deposition in the sham group. However, in the control group, hyperplastic collagen fibers were easily detected. The amount of collagen deposition detectably decreased in the adhesion tissue in the PE groups. A minimal amount of collagen deposition and a relatively intact peritoneal architecture were detected in the H-PE group. Collagen deposition in the L-PE group was less than that in the HA group. Furthermore, the adhesions thickness results were equivalent (Figure 6B).

**Figure 7:**
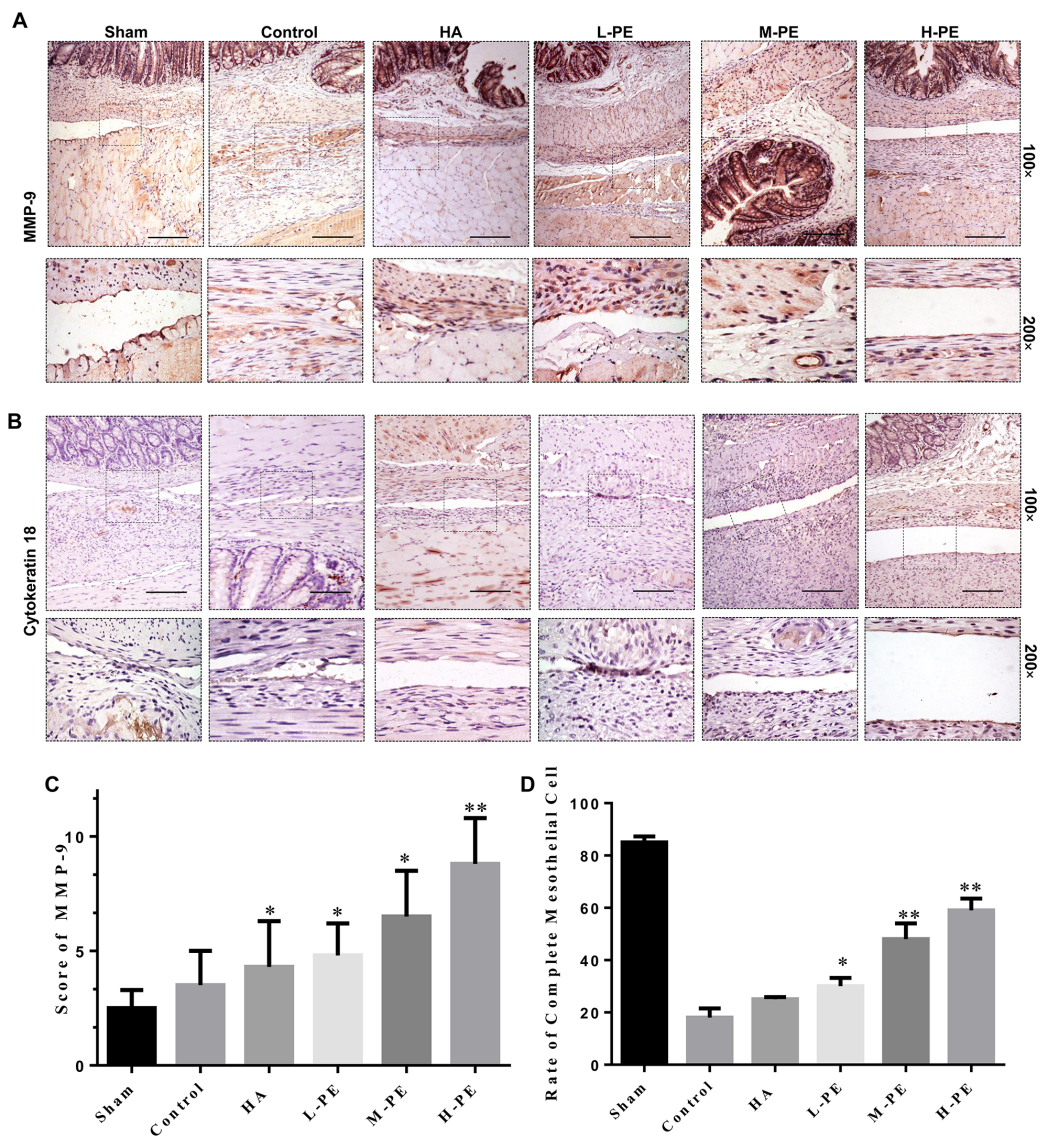
Immunohistochemical staining of MMP-9 and cytokeratin in the postoperative peritoneal adhesions or the damaged areas on the opposing parietal peritoneum in each group of rats (100× magnification in the upper row, and 200× magnification in the bottom row) With the exception of the sham group, the adhesion severity was lower, the MMP-9 expression was higher, and the collagen degradation was improved compared to the control group **(A)**. In **(B)**: in the sham operation group, the epithelial cells were in a complete, continuous line with almost no brown stain, indicating that the mesothelial cell layer was unscathed). In the control group, the brown thin continuous line was completely interrupted; the interrupted brown line indicates an injured mesothelial cell layer. In the HA group, incomplete continuous brown lines were observed, indicating that the peritoneal mesothelial cell layer was not completely repaired by regeneration. In the PE groups, the L-PE performance was similar to that of the HA group, and in the M-PE and H-PE groups, continuous brown lines were observed, indicating that the peritoneal mesothelial cell layer was completely repaired by regeneration. A thin continuous brown line was formed, indicating that the peritoneal mesothelial cell layer was complete. The MMP-9 staining score in the different groups **(C)**. The rate of complete mesothelial cells **(D)**. ^*^ Relative to the control group, *P*<0.05 and ^**^P<0.001.

To further understand the degree of the degradation of the extracellular matrix, we evaluated MMP-9 expression by immunohistochemical staining (Figure 7A) and western blotting (Figure 4). The result showed that MMP-9 expression in the H-PE group was the highest. The expression of MMP-9 was associated with increasing doses of the drug (*P*<0.05) (Figure 7C). It was difficult to distinguish the difference between the HA and L-PE groups, and the sham operation group exhibited the lowest expression level. Therefore, these results indicate that PE treatment can reduce collagen deposition in damaged tissue and adhesion formation in the rat model.

### PE promotes mesothelial cell repair in the damaged peritoneum in the rat model

Mesothelial cell continuity and the repair of the cell layer of the damaged rat peritoneum were further evaluated. The expression of cytokeratin was detected via immunohistochemical staining (Figure 7B). In the sham group, the results showed a complete and continuous cell layer on the surface of the two-layer peritoneum (parietal and visceral). In contrast, relatively few mesothelial cells were present on the surface of the damaged two-layer peritoneum in the control group. In the HA and L-PE group, mesothelial cells were equivalent and inconspicuous. Finally, an abundant and a well-repaired mesothelial layer was apparent in the M-PE and H-PE groups (*P*<0.05) (Figure 7D). In the H-PE group, mesothelial cell repair was more complete and continuous than in the M-PE group. Thus, in our research, PE treatment appeared to contribute to peritoneal mesothelial cellular repair.

## DISCUSSION

In the present study, we investigated the anti-oxidative stress and anti-inflammatory effects of PE on postoperative peritoneal adhesions in an experimental rat model. Our research indicates that PE offers significant protective effects against postoperative peritoneal adhesions through the reduction of inflammation [23] and oxidative stress [24]. Therefore, PE may be useful in the prevention and treatment of peritoneal adhesions.

To date, the most widely accepted method for the prevention of postoperative peritoneal adhesion is to place absorbable barrier or gel between the injured peritoneal surfaces. Studies have shown that HA, chitosan and polyethylene glycol/polylactic acid films reduce adhesions [25–27]. Currently, HA is an anti-adhesion agent that is widely used in clinical practice [28]. However, the anti-adhesion effect of HA is far from satisfactory. Therefore, the effectiveness of PE on preventing adhesion was compared to that of HA in our study.

The generation and elimination of ROS are in a state of dynamic homeostasis when the body is in under normal conditions. The oxidant and anti-oxidant imbalance plays a critical role in pulmonary fibrosis models and fibrotic diseases [29]. ROS is an oxidant that can activate genes that regulate fibroblast proliferation and cell fate [30] and thereby promote peritoneal adhesion fibrosis. Under disease or injury conditions, homeostasis is disrupted, and the anti-oxidant system is disabled due to tissue damage [31]. Oxidative stress stimulates the formation of peritoneal adhesion, and it is propitious to prevent the formation of adhesion by inhibiting the oxidative stress pathway [32]. *in vitro* and *in vivo*, PE protects against rheumatoid arthritis and cellular apoptosis via the suppression of oxidative stress [33]. In the present rat model study, treatment with PE decreased ROS and SOD activity in the enterocoelia and increased the expression of SOD in peritoneal adhesion specimens. Therefore, we suggest that PE treatment can prevent the development of postoperative peritoneal adhesions and that this therapeutic effect of PE is due to an elevation in the levels of locoregional SOD.

When the peritoneum is damaged, multiple cytokines are secreted [34], among which TGF-β1 and IL-6 are deemed to be extremely important for fibrogenesis [35]. Therefore, inflammation may augment TGF-β1 and IL-6, promoting the formation of peritoneal adhesion. An increase in TGF-β1 gene expression has been detected in inflamed, damaged arthritis tissue [36]. TGF-β1 is also regarded as the main fibrogenic and collagenic component in the inflammatory macrophages that infiltrate damaged tissue [37]. IL-6 is a profibrotic cytokine produced by B cells. In damaged tissue, IL-6 promotes fibrosis via its ability to induce fibroblast proliferation, alter tissue inhibitor of metalloproteinase (TIMP) and MMP expression and increase collagen deposition [38]. It has been reported that local alterations of MMPs and TIMPs are regulated by TGF-β1 [39] and hypoxia in mesothelial cells. Of the MMPs, MMP-9 is regarded as a marker of fibrosis; the high expression of MMP-9 corresponds to low fibrosis and the degradation of matrix components. Furthermore, MMP-9 is involved in various aspects of inflammation, including the healing and repairing of tissue injury and in remodeling processes [40]. In the present study, the levels of TGF-β1, IL-6 and inflammatory cell infiltrate were clearly lower in the M-PE and H-PE experimental groups than in the sham and control groups. Immunohistochemical and western blotting detection revealed the lower expression of collagen I and α-SMA and the higher expression of MMP-9 in the PE groups than in the control group. We therefore conclude that PE suppresses postoperative peritoneal adhesions by reducing TGF-β1 and IL-6 levels and the associated inflammatory reaction.

In our study, we also investigated COX-2, which has proinflammatory activities. In previous studies [41, 42], the application of a COX-2 inhibitor to arthritic rat models quickly ameliorated paw edema and relieved joint inflammation. In animal models of arthritis, the selective inhibition of COX-2 activity also modulates systemic and local cytokine generation. Moreover, a previous study [15] showed that selective COX-2 inhibitors can be used to prevent intra-abdominal adhesion formation in rat models. IL-6 is one of the cytokines that promotes the development of arthritis. *in vitro* and *in vivo*, systemic IL-6 was spontaneously produced in controls; its expression was rapidly reversed when COX-2 activity was inhibited. Furthermore, the inhibition could markedly reduce inflammatory cell infiltration and the inflammation of arthritis tissue [43]. This result is consistent with the outcomes of previous studies [23, 24], which have shown that PE suppresses arthritis and hepatic reperfusion injury by decreasing COX-2 expression in rat models. Consequently, COX-2 likely contributes to peritoneal adhesion by potentiating the depletion of IL-6. In our results, the expression of IL-6 and COX-2 and the cellular infiltrate in the H-PE group were significantly decreased. The extent of peritoneal adhesions was also less than that of other groups. The inhibition of COX-2 by PE to partially decrease inflammatory cell infiltrates in peritoneal adhesion specimens likely explains the reduction in the levels of IL-6 and COX-2 proteins.

This study provided evidence for the anti-adhesion effects from the systematic function of PE and for the Enhanced Recovery After Surgery (ERAS^®^) concept (colonic surgery) in which patients are encouraged to consume moderate amounts of water and food as soon as possible after surgery. The early water and food intake could promote intestinal motility [44] without any complications [45]. Therefore, we believe that it is safe and feasible to take PE as an anti-adhesion agent via gavage.

This study provides a reference for the clinical treatment of peritoneal adhesions, but it cannot be considered absolutely conclusive because the human body is often compromised by a variety of diseases, the internal environment is more complex than that of the mouse, and there are also individual variations. Additional clinical trials and the elucidation of underlying molecular mechanisms are required to verify the effects of PE on adhesion formation.

We conclude that PE markedly suppressed postoperative peritoneal adhesions in a dose-dependent manner in a rat model. The protective effects of PE in adhesion treatment may be due to anti-oxidative stress and anti-inflammatory mechanisms and the inhibition of COX-2. Accordingly, we suggest that PE could be used as a therapeutic agent for the inhibition of postoperative peritoneal adhesions. However, more in-depth studies are needed before PE can be applied in the prevention of postoperative peritoneal adhesions in patients.

## MATERIALS AND METHODS

### Experimental animals

Forty-eight Sprague-Dawley male rats (weight, 180-200 g) were obtained from the Animal Resource Center of Xi’an Jiaotong University. The rats were kept in cages under standard conditions and were allowed free access to food and water. The guidelines of the Animal Care and Use Committee of the Xi’an Jiaotong University Health Science Center served as the reference standard. Our experimental procedures and processes were approved by the Ethics Committee.

### Model establishment

The rats were randomly divided into six groups as follows. The animals were anesthetized with a 50 mg/kg peritoneal injection of pentobarbital for all procedures. Prior to incision, the abdomen was shaved, and the skin was sterilized with antiseptics. An approximately 2 cm incision of the abdominal wall was made for each animal. The anterior cecal surface was gently abraded 30 times to the same extent with a wet swab until partial petechial hemorrhages were generated. The abdominal wall that faced the treated cecum was damaged using a medical electric scalpel over an area of approximately 2 cm^2^. The cecum was then placed back in its original location. In the sham operation group, cecal abrasion was not performed, and the abdominal wall was not damaged. Our adhesion model was similar to the one previously reported [15], which mimics the operative process with fewer complications. In the Hyaluronan (HA) group, 1 mL of HA gel was applied on two sides of the trauma and its surrounding area. The three PE groups received low-dose PE (dissolved in normal saline; purity >98%, Sigma-Aldrich Co. LLC., St. Louis, MO, USA) at 10 mg/kg (L-PE); moderate-dose PE at 20 mg/kg (M-PE) or high-dose PE at 40 mg/kg (H-PE) via gavage for 7 days. The sham, control and HA groups were given the same amount of normal saline by gavage for 7 days. The abdominal incision was closed in two layers with a continuous 3/0 silk suture.

### Measurements of the grades of adhesions

After 7 days, a U-shaped incision was made to estimate the adhesions. Subsequently, two independent observers scored adhesion formation in each group of rats blindly in triplicate. The adhesion scoring systems were applied to evaluate both the degree of severity [46] and the extent [47, 48] of adhesion (Supplementary Tables 1 and 2). The overall adhesion score for each animal was the total of the extent score and the severity score. The treatment groups were compared with the control group, and *P*<0.05 indicated a statistically significant difference. After the adhesions were scored, tissue specimens (adhesion tissue, injured cecum wall and parietal peritoneum) and arterial blood samples were collected for the following analyses.

### Histological study

Hematoxylin and eosin (HE) staining was performed for the microscopic histological grading of inflammation. The descriptive system of histology was used to grade the healing process, inflammatory cell reaction, fibroblast activity and neocapillary formation. The system standard [49] is presented in Supplementary Table 3.

### Immunohistochemical analysis

The specimens were removed from -80°C conditions, embedded in paraffin, and cut into 5 consecutive 4-μm thick sections. After deparaffinization in an oven at 60°C for 3 h, the sections were rehydrated with 2 and 3 changes of xylene and ethanol, respectively. A microwave was used for antigen retrieval. The endogenous peroxide activity was quenched with 3% hydrogen peroxide for 20 min at room temperature, and the sections were then rinsed with flowing water 3 times for 5 min per rinse. Nonspecific binding was blocked by BCA (Maixin Biotech. Co., Ltd, Fuzhou, China) goat serum for 20 min at room temperature according to the manufacturer’s instructions. Sections were incubated with rat monoclonal antibodies that targeted collagen-1 (SAB4200678, Sigma, Chicago, USA, 1:200 dilution), superoxide dismutase-2 (SOD-2, SAB2702311, Abcam,Cambridge, UK, 1:200 dilution), cytokeratin 18 (ab27553, Abcam, Cambridge, UK, 1:300 dilution), and α-SMA (sc-53015, Santa Cruz Biotechnology, 1:300 dilution) at 4°C overnight. Then, the sections were incubated in secondary anti-rat antibodies (Santa Cruz Biotechnology, Inc.) for 35 min at 37°C. After washing with PBS, the sections were stained using a DAB Substrate Kit (Thermo Fisher Scientific, Waltham, USA) to visualize the antigen-antibody complex.

To evaluate the staining index, a minimum of five randomly chosen high-power fields were inspected for each section using the scoring system (Supplementary Table 4). The total staining scores (0-12) were determined by multiplying by the score for staining intensity with the score for positive area. The cytokeratin level was evaluated by the average rate of complete and stained mesothelial cells in the selected fields.

### Western blotting analysis

After treatment for 7 days, 20 mg specimens were obtained and incubated on ice with 150 μl tissue lysis buffer (Thermo Fisher Scientific). After 1 h, the homogenate was centrifuged at 800×g for 15 min at 4°C, and the supernatant protein concentration was determined via BAC methods with a protein extraction kit (Fermentas, Beijing, China). We loaded uniform samples of 40 μg of protein onto the sodium dodecyl sulfate-polyacrylamide gels and transferred gels to polyvinylidene fluoride membranes (Millipore, Billerica, MA, USA). The following antibodies were used for Western blotting analysis in our study: anti-COX-2 (Santa Cruz Biotechnology Inc., Dallas, TX, USA; 1:800 dilution), anti-SOD-2 (SAB2702311, Abcam, Cambridge, UK, 1:500 dilution), anti-α-SMA (sc-53015, Santa Cruz Biotechnology, 1:1,000 dilution), anti-MMP-9 (SAB5300247, Sigma, Chicago, USA, 1:800 dilution), and anti-β-actin (Santa Cruz Biotechnology, 1:5,000 dilution). The samples were incubated for 12 h at 4°C. Membranes were incubated with the secondary rat antibodies at 37°C for 2 h. Band intensities were measured with Image-Pro Plus software (v. 5.0; Media Cybernetics, Inc., Rockville, MD, USA).

### Collagen deposition detected via picrosirius red staining

A 0.1% picrosirius red solution (Direct Red 80; Sigma-Aldrich Co) was used to stain collagen. Weigert’s hematoxylin (Wako Pure Chemical Industries, Ltd., Osaka, Japan) was applied as a counterstain. The number of positively stained areas was detected via the microscope (Olympus, MD, Japan) in eight randomly selected fields, and the mean of the eight values was considered the collagen thickness in the adhesions.

### ELISA analysis

Arterial blood was collected into evacuated tubes, and coagulation was promoted. The tubes were then centrifuged at 13,000×g for 15 min at 4°C, and the supernatant was preserved in a -80°C refrigerator for biochemical analysis. The levels of transforming growth factor-β1 (TGF-β1) and IL-6 in serum were tested with commercial ELISA kits (Huamei Biological Technology Co., Ltd., Wuhan, PRC eBioscience) according to the instruction manuals.

### SOD activity and ROS measurement

Enterocoelic fluid specimens were acquired from the abdomens of each animal and then centrifuged at 3,000×g for 30 min [50]. The supernatant was stored at -20°C for tests. The levels of SOD (A001; Nanjing Jiancheng Bioengineering Institute, Nanjing, China) and ROS (E004; Nanjing Jiancheng Bioengineering Institute, Nanjing, China) were measured. The assay was conducted according to the manufacturer’s instructions.

### Statistical analysis

Statistical analyses were performed using SPSS 18.0 (SPSS Inc., Chicago, IL). Values are described as the mean ± standard deviation. The Mann-Whitney U analysis was used to compare adhesion formation in the two groups. The mean values of groups were analyzed via one-way analysis of variance (ANOVA). All differences were considered to be statistically significant at a *P* value of <0.05.

## SUPPLEMENTARY MATERIALS FIGURE AND TABLES


