# Supplementary material for: Paeoniflorin prevents postoperative peritoneal adhesion formation in an experimental rat model
Source: Oncotarget. 2017 Sep 28;8(55):93899–911. doi: 10.18632/oncotarget.21333 (PMC5706843; doi:10.18632/oncotarget.21333)
Supplement: Supplementary file 1 [file oncotarget-08-93899-s001.pdf]

Paeoniflorin prevents postoperative peritoneal adhesion formation in an experimental rat model

SUPPLEMENTARY MATERIALS

Supplementary Table 1: Severity of adhesions among the six groups (n)

Groups [N=8]	Macroscopic severity of adhesion score				P-value
	0	1	2	3	
Sham	6	2	0	0	-
Control	0	0	4	4	-
HA	1	2	2	3	0.000
L-PE	0	2	3	3	0.000
M-PE	2	4	1	1	0.027
H-PE	4	2	2	1	0.016

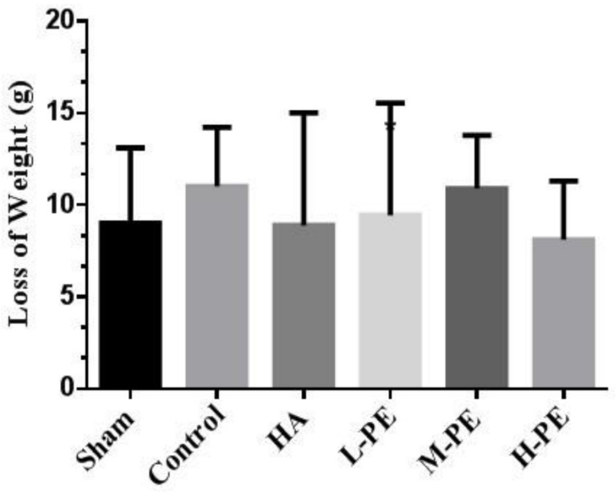

Supplementary Figure 1: Preoperative and postoperative changes in the body weight of animals were not significantly different among the groups.

Supplementary Table 2: Extent of adhesions among the six groups (n)

Groups [N=8]	Macroscopic extent of adhesion score				P-value
	0	1	2	3	
Sham	6	2	0	0	-
Control	0	0	4	4	-
HA	1	1	3	3	0.000
L-PE	0	2	4	2	0.025
M-PE	2	4	2	0	0.025
H-PE	4	3	1	0	0.027

Supplementary Table 3: Parameters of histopathologic examinations

Four aspects	Parameters*		
	1	2	3
Mucosal healing	no epithelialization	attempt at epithelialization	glandular formation
Inflammatory cell exudate	heavy infiltration	moderate infiltration	mild infiltration
Fibroblastic activity	mild infiltration	moderate infiltration	heavy infiltration
Neo-capillary formation	mild infiltration	moderate infiltration	heavy infiltration

*Each of the items contributed to a relevant score; for example, if the specimen had characteristics of no epithelialization, heavy infiltration, mild infiltration and mild infiltration, it got a score of 4. The four items of description were the subsets of mucosal healing, inflammatory cell exudate, fibroblastic activity and neo-capillary formation respectively.

Supplementary Table 4: The immunohistochemical scoring system

Two aspects	Scores				
	0	1	2	3	4
Intensity	Negative	Weak	Moderate	Strong	-
Frequency of Positive Cells	<5%	5% to 25%	6% to 50%	51% to 75%	> 75%
